# Effect of Young’s Modulus and Surface Roughness on the Inter-Particle Friction of Granular Materials

**DOI:** 10.3390/ma11020217

**Published:** 2018-01-31

**Authors:** Chitta Sai Sandeep, Kostas Senetakis

**Affiliations:** Department of Architecture and Civil Engineering, City University of Hong Kong, Kowloon Tong, Hong Kong, China; sschitta2-c@my.cityu.edu.hk

**Keywords:** friction, contact mechanics, granular materials, roughness

## Abstract

In the study we experimentally examine the influence of elastic properties and surface morphology on the inter-particle friction of natural soil grains. The experiments are conducted with a custom-built micromechanical apparatus and the database is enhanced by testing engineered-reference grains. Naturally-occurring geological materials are characterized by a wide spectrum of mechanical properties (e.g., Young’s modulus) and surface morphology (e.g., roughness), whereas engineered grains have much more consistent characteristics. Comparing to engineered materials, geological materials are found to display more pronounced initial plastic behavior during compression. Under the low normal load range applied in the study, between 1 and 5 N, we found that the frictional force is linearly correlated with the applied normal load, but we acknowledge that the data are found more scattered for natural soil grains, especially for rough and weathered materials which have inconsistent characteristics. The inter-particle coefficient of friction is found to be inversely correlated with the Young’s modulus and the surface roughness. These findings are important in geophysical and petroleum engineering contents, since a number of applications, such as landslides and granular flows, hydraulic fracturing using proppants, and weathering process of cliffs, among others, can be simulated using discrete numerical methods. These methods employ contact mechanics properties at the grain scale and the inter-particle friction is one of these critical components. It is stressed in our study that friction is well correlated with the elastic and morphological characteristics of the grains.

## 1. Introduction

Understanding of the mechanical behavior at the micro-scale is needed to comprehend the complex macro-scale behavior of geological materials. Normal and tangential loading behavior, inter-particle friction and stiffness are some of the key parameters needed as input in numerical modeling of various applications within geotechnical-geological engineering contents. In recent years, advancements in the discrete element modeling (DEM) [1] have provided useful insights into the understanding of the physics and mechanics of granular materials. Applications to geophysical, geological, petroleum, and geotechnical engineering, as well as other applications related to granular flows and powders, have further stressed the necessity to explore the contact mechanics properties of naturally-occurring materials in the laboratory. For engineered materials, several researchers have found that the inter-particle (or inter-face) friction is mainly influenced by the surface roughness and the Young’s modulus of the contacting surfaces [2,3,4,5,6]. Nonetheless, compared to engineered materials, a limited number of experimental studies have been carried out on the contact mechanics and frictional behavior of geological materials in the last few decades, especially for the grain-grain type of contact [7,8,9,10,11,12,13,14,15,16,17,18].

Unlike artificial materials, the roughness and overall morphology of soil particles are characterized by relatively high discrepancies and they depend on the type of the parent rock and the various environmental conditions (e.g., weathering, transportation, and depositional processes) they are subjected to. For rocks, researchers have found a strong effect of surface roughness on the friction at low confining pressures [19]. Some of the recent studies of geological materials [14,18,20,21,22,23] have shown significant different frictional characteristics between variable types of soil grains. This has been attributed, partly, to variabilities in surface roughness characteristics, but more thorough discussions into the coupled effect of surface geometrical features and material elastic properties have not been considered. It has been highlighted, through DEM simulations, that the inter-particle friction may have a critical role on the mechanical behavior of granular assemblies [24,25,26]. Tangential contact models, typically employed in DEM analysis, for example the widely-known models proposed by Mindlin and Deresiewicz [27] or Thornton and Yin [28] use, as input, the inter-particle coefficient of friction in their expressions correlating tangential (or frictional) force to sliding displacement. Thus, it is stressed that proper understanding of the factors controlling friction must be obtained and incorporated in the numerical simulation of variable problems involving granular matter. 

In the broader fields of tribology and materials engineering, there has been significant progress over the previous years on the frictional behavior of interfaces. Researchers such as Kogut and Etsion [29], Bhushan et al. [30] and Greenwood and Williamson [31] have found that the frictional force (*F_F_*) depends on the applied normal force (*F_N_*) and the area of contact (*A*). For multi-asperity contact surfaces, Greenwood [32] mentioned that the inter-particle friction (*μ*) is a function of material Young’s modulus (*E*) and surface roughness. Chang et al. [33] have shown that, for machine surfaces, μ depends on the material properties, the surface topography and the normal load magnitude. Bowden and Tabor [34] proposed that *F_F_* is dependent on *A* via two basic mechanisms, shearing and ploughing, and that for softer materials in contact it is expected that A will be greater. Hence, the value of *A* is a function of the applied normal force, the Young’s modulus, and the surface roughness of the materials in contact. The value of *A* can be obtained by using various models [35]. The models proposed by Hertz [36] and Greenwood [32] are the most commonly used to back-calculate the area of contact. In contrast to manufactured (or engineered) materials, natural soil grains are characterized by a wide spectrum of surface geometrical features, i.e., a great variability in surface roughness and shape outlines, as well as a broad range of elastic properties dependent upon the mineral composition and geological-environmental processes they have been subjected to. 

In this paper, we have studied the combined effect of surface roughness and Young’s modulus on the inter-particle friction for geological materials along with engineered materials to obtain some general trends of behavior. We found that all the materials showed some initial soft behavior during normal loading which was more pronounced for natural soil grains, especially those being subjected to weathering or having higher roughness. We present herein that, for all the materials used in this study, the frictional force, defined after a first hardening regime, varies linearly with the applied normal force. Furthermore, we show that the inter-particle friction for both engineered materials and natural soil grains is a function of material Young’s modulus and surface roughness.

## 2. Materials

The micro-mechanical behavior of a variety of grains was examined in the laboratory using a new-generation custom-built inter-particle loading apparatus developed at the City University of Hong Kong. These materials included chrome steel balls (CSB) and glass balls (GB) as engineered grains, Leighton Buzzard sand (LBS), which is a silica-rich sand of relatively smooth and hard surface grains, limestone (LS), which is a sand of biogenic origin with softer and rougher surface, and completely decomposed granite (CDG). The latter material, which is abundant in tropical/sub-tropical regions, consists of heavily decomposed grains, due to, majorly, chemical weathering, which has altered some of its major minerals such as feldspars and mica to a heavy coating of clayey-platy grains resulting in highly rough, but relatively soft, surfaces. These broad range of materials are commonly encountered in geotechnical-geological engineering practice and they comprise representative types of grains to obtain insights into the frictional behavior of granular materials (covering a broad range of surface roughness and Young’s modulus). Quartz type grains, such as LBS, may find critical applications as proppant in hydraulic fracturing, with significant interest in petroleum engineering [20]. In geophysical-geological research and practice, soil and rock mass movements (e.g., landslides) are commonly studied numerically within the content of granular flows. Such problems may involve a broad range of materials, thus this work gives some upper and lower bounds of behavior examining a wide range of grains of variable elastic and morphological characteristics. Some of these materials were studied individually in previous works [13,17,23], but an overall view of the significant factors affecting the friction was not attempted in a systematic manner before.

The material properties are reported in Table 1. The commercially-available CSB and GB grains which are 2 mm in diameter are tested along with geological materials, with the latter being mechanically sieved, and sizes ranging from 1.18 to 3.00 mm are chosen for this study. This size range was chosen because of limitations of the apparatus and testing techniques in sample preparation [37], even though, this size range is representative of geological materials (e.g., sand-sized grains) encountered in many applications. In Table 1, S and R correspond to the grain shape descriptors of sphericity and roundness, respectively, which are obtained from visual observation of a representative set of grains by using an empirical chart proposed by Krumbein and Sloss [38]. The surface roughness of the materials is obtained by using the optical surface profiler of the City University of Hong Kong. An area of 20 × 20 µm is taken at a magnification of 100×, while the effect of the curvature is removed (i.e., the grain surface is flattened via an option of the software of the profiler), and the computed surface roughness is presented in terms of the root mean square, RMS, roughness (denoted as *α*). The values of the Poisson’s ratio (*υ*) for the different material types are taken from the literature. All these values are summarized in Table 1.

## 3. Experimental Equipment and Methods

**Figure 1:**
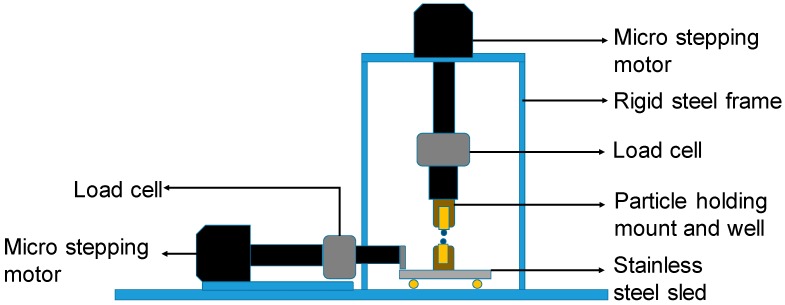
Schematic diagram of the inter-particle loading apparatus of the City University of Hong Kong.

**Figure 2:**
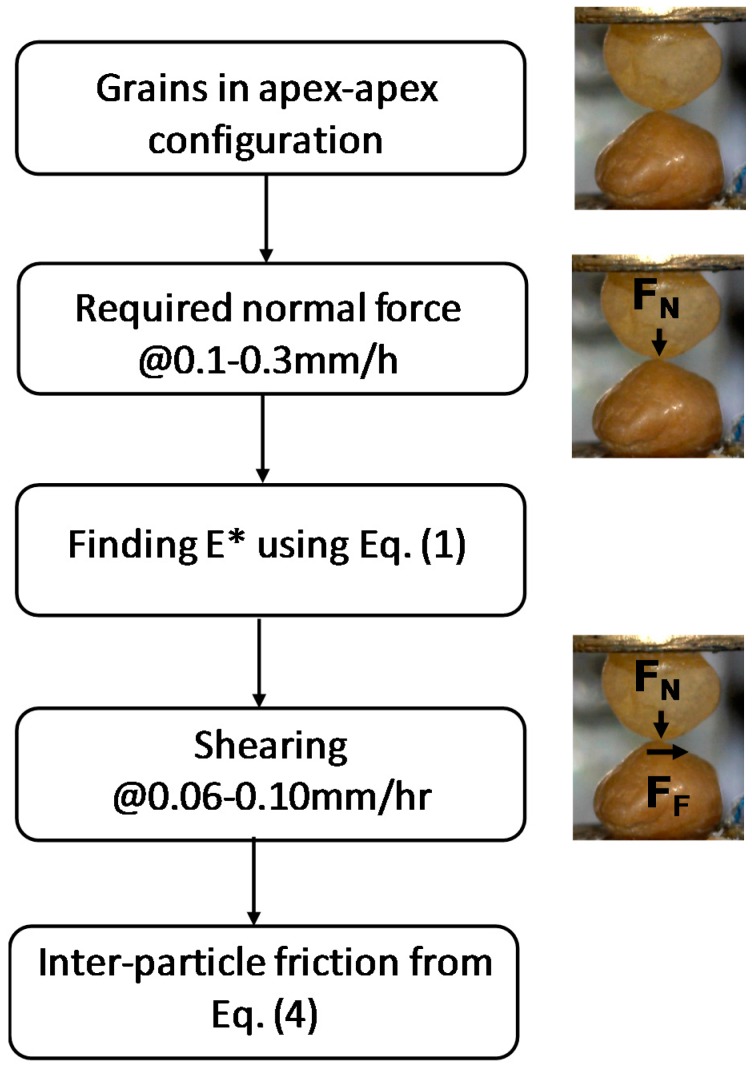
Flowchart showing the methodology used to derive inter-particle friction from present experiments.

The schematic view of the inter-particle loading apparatus used in the study is shown in Figure 1 [17,37]. It consists of a stiff frame attached to the base and it is capable of applying/measuring forces and displacements at the contacts of sand-sized grains in the vertical and two orthogonal horizontal directions. Each arm consists of a micro-stepping motor, a load cell of resolution of 0.02 N, and various other mechanical parts. The displacements are measured by using non-contact eddy sensors with a high resolution of 10^−5^ mm and the quality of the signal output is improved by using high-performance data logging and filter systems. The particles are glued to mounts and left to dry for 24 h before being placed into the wells. The bottom well is placed on the sled and the top well is attached to the vertical arm. The naturally-occurring soil grains used in this study are not generally spherical. Thus, the particles are aligned in an apex to apex configuration by using two digital micro-cameras which are placed in two orthogonal directions. The methodology used to derive the inter-particle friction from the present experiments is shown in Figure 2. 

The micro-mechanical testing program was conducted at a temperature of 22–25 °C and a relative humidity of 60%. The normal force (*F_N_*) is applied by moving the top particle towards the bottom one by using the vertical arm at a displacement rate of 0.1–0.3 mm/h. In this testing program, the application of *F_N_* is limited to up to 5 N. It has been shown from numerical simulations [43] that the normal force magnitude developed at the soil grain contacts within the content of typical geotechnical-geological engineering examples may range up to 4–5 N. Previous works by the authors [22,23] showed that a plastic deformation might occur during shearing if the normal loads exceed 5 N for typical quartz sand grains. Thus, an intention of this work was to study the frictional behavior of soil and engineered grains within this typical normal force range (1–5 N) avoiding excessive surface damage of natural soil grains in shearing mode.

## 4. Results

**Figure 3:**
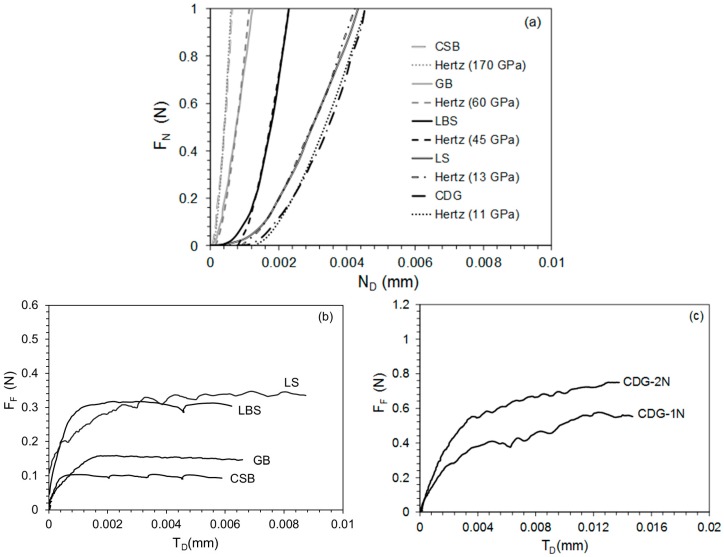
Representative curves showing: (**a**) normal force against normal displacement up to 1 N along with Hertzian fitting; (**b**) frictional force against displacement for materials tested at 1 N of normal force; and (**c**) frictional force against displacement for CDG at 1 and 2 N of normal force.

Figure 3a reveals the normal force-displacement behavior of representative tests covering the broader range of the examined materials. It can be observed that during the compression of the grains, an initial soft response is occurred, wherein this soft behavior is more evident for geological materials compared to engineered materials, particularly for those having higher roughness and/or being subjected to weathering (e.g., LS and CDG). These variabilities in grains, apart from the influence of the surface geometrical features, might be advanced by the variabilities in material Young’s modulus and the subsequent plastic deformation of asperities, as also pointed out by Sandeep and Senetakis [23]. The Hertzian expression [36] is fitted to the normal force-displacement curve using Equation (1):

(1) $$F_{N}=\frac{4}{3}{\left(R^{*}\right)}^{\frac{1}{2}}\left(E^{*}\right){\left(N_{D}\right)}^{\frac{3}{2}}$$

where *N_D_* is the normal displacement, *F_N_* is the normal force corresponding to *N_D_*, R* is the equivalent particle radius of both top and bottom grains calculated from Equation (2), and E* is the equivalent Young’s modulus which is obtained from Equation (3):

(2) $$\frac{1}{R^{*}}=\frac{1}{R_{1}}+\frac{1}{R_{2}}$$

(3) $$\frac{1}{E^{*}}=2\frac{\left(1-\upsilon^{2}\right)}{E}$$

In Equation (2) *R*_1_ and *R*_2_ correspond to the average radius in three dimensions of the top and bottom grains in contact, which is measured by a Vernier caliper. Note that the geological particles used in this study are not perfectly spherical and they are rough, so obtaining the average radius using this method might introduce some error in the calculation of R* and *E*. In Equation (3), *E* corresponds to the apparent Young’s modulus of the grain pair in contact. The apparent Young’s modulus is found using the best fit (Hertzian) curve to the experimental data, which is applicable beyond the first regime of the soft response (in general beyond about 0.5μm for engineered grains, 1 to 2 µm for LBS, and beyond 3 µm for the softer grains of LS and CDG). For representative grains, the average values of the apparent Young’s modulus along with their standard deviation of the materials tested are presented in Table 1. Note that for each pair of grains, for simplicity, the apparent Young’s moduli of top and bottom grains in contact are assumed to be the same. Even though the Hertzian curves reasonably fit the experimental curves, it should be noted that these values of Young’s modulus are apparent only. However, this theoretical fitting, based on Equations (1)–(3), gives a general idea of the materials elastic properties, which can be used for comparison purposes as it is difficult to obtain the accurate elastic properties of naturally-occurring geological materials with variable mineralogy and irregularities. 

After reaching the required *F_N_*, the shearing force (*F_F_*) is applied to the grain contacts by moving the lower grain at a displacement rate of 0.06–0.10 mm/h, while maintaining the constant *F_N_*. Representative curves which demonstrate the highly non-linear increase in *F_F_* with tangential displacement (*T_D_*) at *F_N_* equal to 1 N are shown in Figure 3b,c. The mobilized inter-particle coefficient of friction (*μ*) is computed based on the relationship between *F_F_* and *F_N_* from Equation (4). For further interpretations and correlations herein, the mobilized friction after the occurrence of the hardening regime is used (i.e., reaching the first plateau of the shearing force-displacement curve):

(4) $$\mu =\frac{F_{F}}{F_{N}}$$

An average of ten tangential shearing tests are conducted at values of *F_N_* ranging from 1 to 5 N for each material type to obtain values of *μ*. 

Throughout the total set of ten experiments for each material type, the average values of *μ* and their standard deviations are given in Table 1. It is noticed that the standard deviations of *μ* for geological materials are higher compared to the engineered materials CSB and GB, which might be attributed to the discrepancies in the mechanical properties and morphology between the grain pairs tested (e.g., less consistent surface characteristics and elastic properties for the natural sand grains, especially those from LS and CDG). It is noticed that within the relatively narrow range of sliding velocities applied during the shearing tests, the magnitude of sliding velocity did not produce any notable effect on the resultant inter-particle friction.

**Figure 4:**
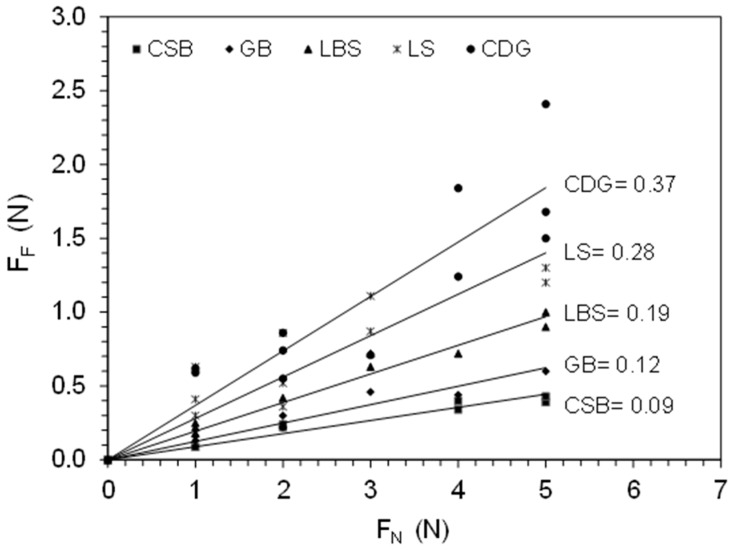
Variation of frictional force with normal force for the materials tested and corresponding inter-particle coefficients of friction.

The envelopes of the different materials, expressed with the values of *F_F_* (vertical axis) against *F_N_* (horizontal axis) are presented in Figure 4. It was found that for the low normal force range in the study, up to 5 N, *F_F_* varies linearly with *F_N_* for all the materials tested. The strongest correlations, expressed with the coefficient of determination (R^2^), were found for the engineered materials GB and CSB, as well as for LBS quartz grains (*R*^2^ ≈ 0.94–0.95 as shown in Table 1). For CDG and LS, *R*^2^ was found equal to 0.81 and 0.77, respectively, which resulted, primarily, from the higher inconsistency of the surface characteristics and back-calculated Young’s modulus (apparent) for these two natural sands. Nevertheless, these observations show that, even for geological materials, the inter-particle friction depends on the contact area, which, in turn, depends on the normal force magnitude.

Based on Hertz [36], the contact area between the two bodies is given by Equation (5):

(5) $$A=\pi {\left[\frac{3R\;F_{N}}{4E^{*}}\right]}^{\frac{2}{3}}$$

We can obtain the value of *F_F_* from Equation (5), as *F_F_* is the product of *A* and the critical shear strength (*τ*). However, Equation (5) is theoretically valid only for single asperity contact. For multi-asperity contact, assuming plastic deformations are absent for loads less than 5 N, Equation (6) presents the correlation between the inter-particle coefficient of friction, surface roughness, and the elastic properties of the surfaces in contact [32]:

(6) $$\mu =\frac{3}{2}\tau {\left[\frac{\pi R^{*}}{2\alpha }\right]}^{\frac{1}{2}}\left[\frac{\left(1-\upsilon^{2}\right)}{E}\right]$$

**Figure 5:**
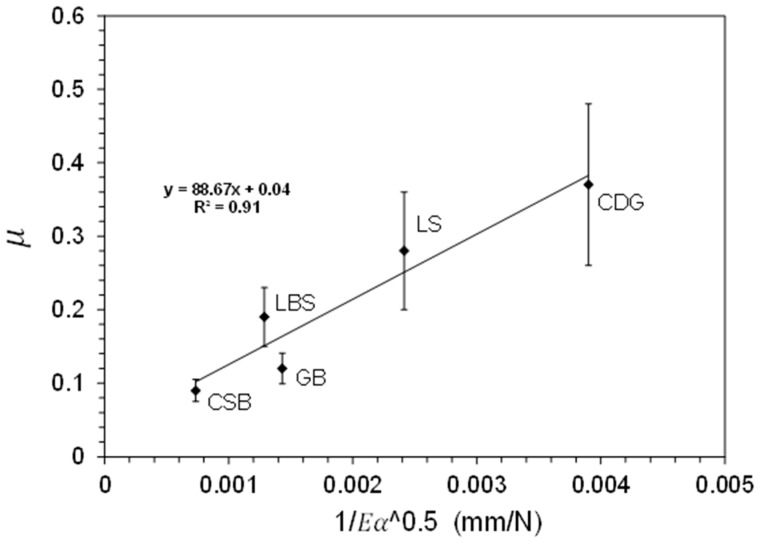
Inter-particle coefficient of friction as a function of Young’s modulus and surface roughness.

The materials are taken nearly of similar size and the *υ* values for different grain types are also nearly the same. Therefore, from Equation (6), the inter-particle friction can be linked to the surface roughness and the Young’s modulus. It can be observed from Table 1, that the inter-particle friction increases with the increase in roughness. However, the wide range of the apparent Young’s moduli of the studied materials can also affect the contact area and the inter-particle friction. Thus, both surface roughness and apparent Young’s modulus play key roles in the frictional behavior of the tested materials. This gives a nice approximation between the combined effect of surface roughness (taken as the square root of α) and the apparent Young’s modulus to the inter-particle friction of the materials tested, as shown in Figure 5.

## 5. Discussion and Conclusions

To obtain a general idea on the effect of the mechanical and surface properties of geological materials on their frictional behavior, we conducted a micromechanical experimental study examining the frictional and normal contact responses of natural soil grains, and we also included engineered materials in our study. The tested materials exhibit a wide range of surface roughness and apparent Young’s modulus and their combined effect on the inter-particle friction can be rationalized as follows. Within the scatter of the data from Figure 5, like engineered materials, the frictional response of geological materials is also sensitive to changes in Young’s modulus and surface roughness. With the increase in the value of the product between *E* (apparent Young’s modulus) and *α* (surface roughness), the value of A is decreased, thereby decreasing the inter-particle friction. This linear fit might pass through *μ* ≅ 0, when the product of *E* and *α* reaches infinite value, which is not possible for naturally-occurring geological materials with a finite range of Young’s modulus (speaking in terms of non-conforming grain-type contacts). If it reaches an infinite value under any condition, Equation (6) is no longer valid. From the materials tested in this program, the CDG (completely decomposed granite) shows the highest values of μ compared to the other materials, which is controlled by the coupled effect of rough surfaces and reduced Young’s modulus, probably related, primarily, with the weathering process the grains have been subjected to. In modeling variable geophysical-geological problems via DEM, a modeler needs to account for the important role of the contact area on the inter-particle friction. The results of Figure 5 can draw some upper-lower bounds of behavior within the broad range of applications in geo-science and engineering of granular materials, primarily those involved in natural processes and problems. 

## Figures and Tables

**Table 1:** Characterization of materials along with results from the inter-particle tests.

Materials	CSB	GB	LBS	LS	CDG
Diameter (mm)	2.00	2.00	1.18–2.36	1.18–3.00	1.18–2.36
Sphericity (S)	1	1	0.8	0.7	0.8
Roundness (R)	1	1	0.7	0.4	0.6
𝛼 (nm)	62 ± 19	145 ± 28	223 ± 61	670 ± 221	1341 ± 390
Poisson’s ratio (υ)	0.30	0.30	0.25	0.30	0.25
Inter-particle friction (*µ*)	0.09 ± 0.02	0.12 ± 0.02	0.19 ± 0.04	0.28 ± 0.08	0.37 ± 0.11
*R*^2^	0.94	0.94	0.95	0.77	0.81
Young’s modulus E (GPa)	173 ± 11	58 ± 7	52 ± 12	16 ± 6	7 ± 3

Poisson’s ratio values based on [39,40,41,42], *R*^2^: Coefficient of determination of *F_F_*-*F_N_* envelopes
